# Implications of DNA damage in chronic lung disease

**DOI:** 10.3389/fcell.2024.1436767

**Published:** 2024-10-31

**Authors:** Dingning Zhang, Tong Sun, Jiahui Bao, Jianhua Fu

**Affiliations:** Department of Pediatrics, Shengjing Hospital of China Medical University, Shenyang, Liaoning, China

**Keywords:** asthma, BPD, COPD, DNA damage, lung disease

## Abstract

DNA plays an indispensable role in ensuring the perpetuation of life and safeguarding the genetic stability of living organisms. The emergence of diseases linked to a wide spectrum of responses to DNA damage has garnered increasing attention within the scientific community. There is growing evidence that patterns of DNA damage response in the lungs are associated with the onset, progression, and treatment of chronic lung diseases such as chronic obstructive pulmonary disease (COPD), asthma, and bronchopulmonary dysplasia (BPD). Currently, some studies have analyzed the mechanisms by which environmental factors induce lung DNA damage. In this article, we summarize inducible factors of lung DNA damage, current indicators, and methods for diagnosing DNA damage in chronic lung diseases and explore repair mechanisms after DNA damage including nonhomologous end-joining and homology-directed repair end joining pathways. Additionally, drug treatments that may reduce DNA damage or promote repair after it occurs in the lungs are briefly described. In general, more accurate assessment of the degree of lung DNA damage caused by various factors is needed to further elucidate the mechanism of lung DNA damage and repair after damage, so as to search for potential therapeutic targets.

## 1 Introduction

The primary aim of life is to transmit genetic material effectively through DNA. Both internal [reactive oxygen species (ROS), reactive nitrogen species (RNS)] and external factors (radiation, UV, chemicals like tobacco smoke’s polycyclic aromatic hydrocarbons) can damage DNA, disrupting cellular balance and affecting health. Events like spontaneous decay, replication errors, and cellular metabolism frequently lead to DNA alterations, such as base loss, modifications, bond breakage, and structural strand breaks. Common DNA lesions comprise spontaneously occurring single-strand breaks (SSBs) and highly hazardous double-strand breaks (DSBs), which are the most harmful DNA lesions in cells (Abbotts and Wilson, 2017).

DNA damage can result in abnormal cellular function and genetic mutations that contribute to the progression of diseases. For instance, in COPD, DNA damage can lead to lung cell senescence and apoptosis, exacerbating airway remodeling and im-pairing lung function (Collaborators GBDCRD, 2017; Ritchie and Wedzicha, 2020; Barnes, 2020; Giuranno et al., 2024; Kuwano et al., 1996; Yao and Rahman, 2012; Adeloye et al., 2022). In asthma, DNA damage can impact the barrier function and immunomodulatory role of airway epithelial cells, thereby worsening airway inflammation and hyperresponsiveness (Allbright et al., 2024; Lambrecht and Hammad, 2015; Zheng et al., 2024; Ba et al., 2015). Research suggests that bronchopulmonary dysplasia (BPD) is a common condition resulting from inadequate lung development in infants born prematurely before 32 weeks of gestation. Additionally, DNA damage can hinder normal lung development by affecting the proliferation and differentiation of lung stem cells (Reyburn et al., 2012; Tong et al., 2023). Due to the high incidence and harmfulness of chronic lung disease, researchers continue to investigate and have discovered that DNA damage can modify the expression and regulation of genes associated with chronic lung disease, resulting in airway remodeling and heightened susceptibility and severity (Yousefi et al., 2018; Aguilera-Aguirre et al., 2015).

The DNA damage response (DDR) is an innate surveillance system that monitors genome integrity amidst factors affecting genetic stability. It reacts according to the specific lesion detected, transmitting information to pathways regulating cell cycle progression. The double-strand break repair pathway is one of the mechanisms employed for DNA damage repair (Carusillo and Mussolino, 2020). Double-strand breaks are considered the most critical and hazardous form of DNA lesions, as their failure to be repaired can result in cell death. To ensure gene integrity, mammalian cells have developed diverse strategies for mending double-strand breaks, including nonhomologous end-joining (NHEJ) and homology-directed repair (HDR) (Carusillo and Mussolino, 2020). Repair mechanisms employed by different organisms and organ diseases vary.

This review thoroughly explores DNA damage in chronic respiratory diseases (COPD, asthma, BPD), while also including markers, repair mechanisms, and treatment strategies.

## 2 Mechanisms of DNA damage occur in chronic respiratory diseases

### 2.1 Environmental factors and DNA damage in chronic lung disease

Exogenous environmental factors such as cigarette smoke (CS), ambient air pollution PM (PM10 and PM2.5), house dust mites (HDM), inhalable quartz, metal powders, mineral asbestos fibers, O_3_, soot from gasoline and diesel engines, and ionizing radiation cause lung DNA damage, which can result in lung disease by altering the body’s ROS levels, affecting lipids and proteins, and disrupting various cellular processes, ultimately leading to an imbalance between oxidation and antioxidant systems (Yuan et al., 2020; Valavanidis et al., 2013; Chan et al., 2016). COPD is primarily caused by CS exposure. Researchers have observed an increase in the levels of markers associated with DNA damage in the plasma and serum of individuals with COPD. Furthermore, the activation of DNA DSBs triggers the cyclic GAS/STING pathway, resulting in the secretion of type I interferons and subsequent lung inflammation (Giordano et al., 2022; Nascimento et al., 2019). DNA damage was notably increased in patients with CS and biomass fuel induced COPD; the impact was particularly pronounced among individuals diagnosed with smoking-related COPD (Ceylan et al., 2006). Notably, individuals with asthma who smoke exhibit oxidative DNA damage in lung cells, ultimately resulting in apoptosis (Proklou et al., 2013).

**FIGURE 1:**
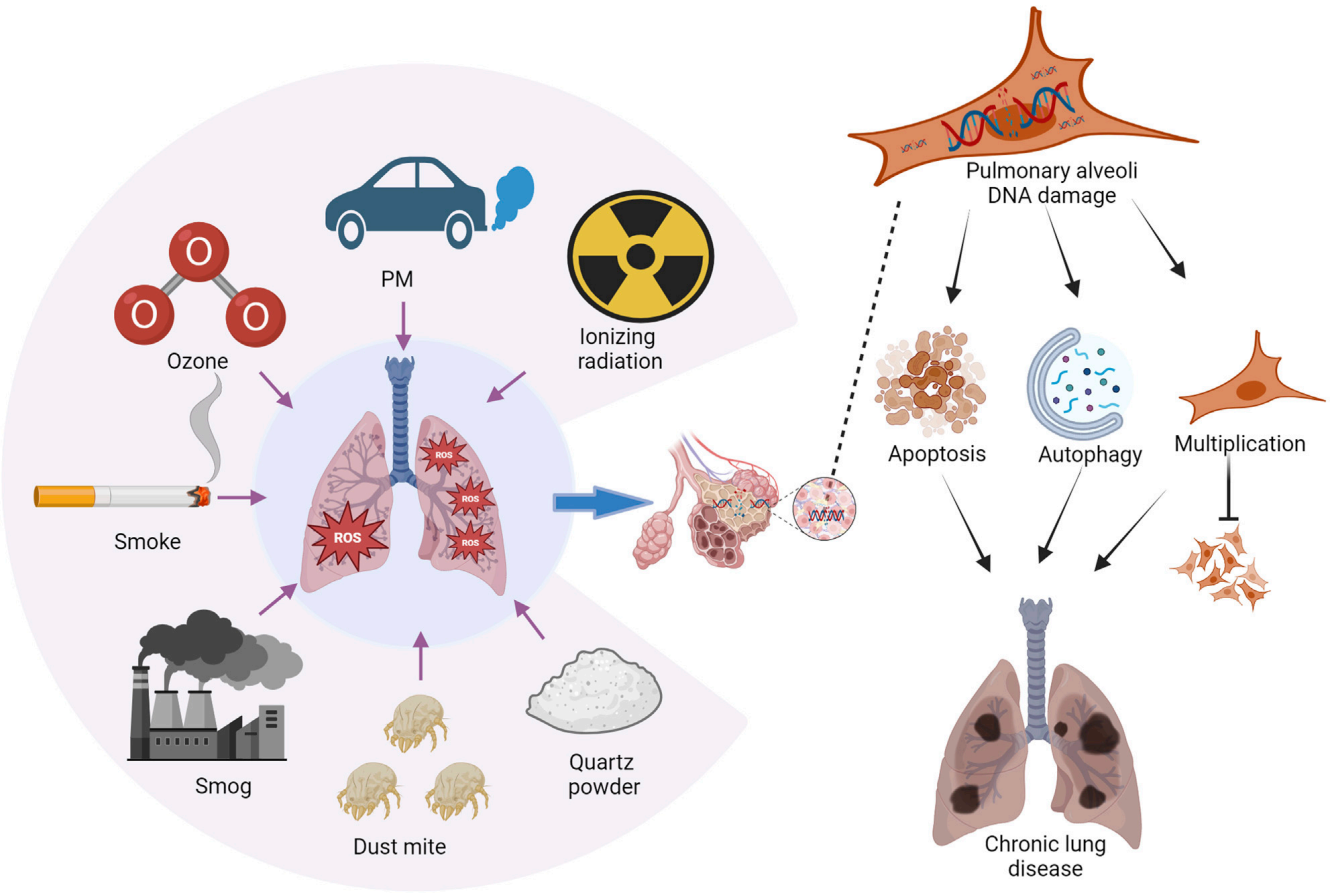
Environmental factors contributing to chronic lung disease. Chronic lung disease is influenced by exposure to various environmental substances, including tobacco smoke, vehicle emissions, fine particulate matter (PM2.5), O_3_ pollution, HDM, quartz and metal powders, and ionizing radiation. Exposure to these elements can lead to the accumulation of reactive oxygen species (ROS) in the lungs. Consequently, this may result in the apoptosis and autophagy of lung cells, along with proliferation disorders that ultimately contribute to lung injury. Image generated using Bio-Render.

Apart from CS, exposure to ambient air pollutants like PM2.5 boosts ROS production in the body, overwhelming the lung’s antioxidant system and causing airway hyperreactivity and aggravated inflammation, ultimately contributing to asthma (Liu et al., 2022). HDM and O_3_ have been shown to induce asthma via DNA damage. O_3_ concentration plays a crucial role in determining the kinetics of activation and deactivation of DNA damage (Liu et al., 2022; Xu et al., 2022; Kozumbo and Agarwal, 1990). Additionally, lung samples collected from individuals diagnosed with asthma revealed the presence of DSBs (Carusillo and Mussolino, 2020). Exposure to high oxygen levels induces the accumulation of DSBs and halts the cell cycle, causing oxidative DNA damage in the lung epithelial cells. Moreover, 8-hydroxy-2′-deoxyguanosine (8-OHdG) is a widely recognized indicator of oxidative DNA damage, which leads to pulmonary injury and BPD onset (Tong et al., 2023; Jin et al., 2015) (Figure 1).

### 2.2 Oxidative stress and DNA damage in chronic lung disease

In chronic respiratory diseases, the inflammatory responses can result in an elevated production of reactive oxygen species (ROS), encompassing superoxide anions, hydrogen peroxide, and highly reactive hydroxyl radicals (Sul et al., 2023; Boukhenouna et al., 2018; Seimetz et al., 2020). ROS possess the ability to directly damage DNA molecules, leading to DNA strand breakage, base oxidation modification, and other forms of damage. For instance, hydroxyl radicals have the capability to react with guanine within DNA and generate 8-OHdG, which serves as a prevalent indicator of oxidative damage (Shigenaga and Ames, 1991).

### 2.3 Inflammatory mediators and DNA damage in chronic lung disease

In chronic respiratory diseases, inflammatory cells release various inflammatory mediators, such as cytokines and chemokines, which can impact cellular metabolism and signaling pathways, ultimately leading to DNA damage. The increased levels of TNF-α, IL-1β, and TGF-β1 in COPD rats exacerbate inflammation, while the levels of IL-4, IL-5, IL-13, IL-12, and IFN-γ are elevated. These mediators activate oxidative stress response and generate excessive ROS, resulting in DNA damage within the lungs (Li et al., 2014; Zhang et al., 2016; Tian et al., 2024; Geraets et al., 2007). In asthma cases, lipid mediators like cysteinyl leukotrienes promote activation and pulmonary inflammation through binding of leukotriene C4 (LTC4) to its internalizing receptors. This leads to nuclear and perinuclear translocations of NOX4 that initiate ROS production along with oxidative DNA damage, apoptosis, and necrosis (Dvash et al., 2015; Lund et al., 2017). Inflammatory mediators also play a crucial role in the severity of BPD. The researchers discovered that the hyperoxia-induced preterm baboon BPD model cases, caseinase-1 activity is increased which upregulates the expression of IL-1β and IL-18 causing an increase in cell death incidence leading to alveolar inflammation and abnormal alveolar simplification (Liao et al., 2015). Nuclear factor E2–related factor 2 (Nrf2) plays a pivotal role in cellular resistance against oxidative stress injury. Imbalance in Nrf2 results in oxidative stress damage that increases inflammatory activation as well as DNA damage potentially halting alveolar development among preterm infants under high oxygen conditions (Wang et al., 2023).

## 3 DNA damage markers from various sources in chronic lung disease

Chronic lung disease can impact DNA damage through various mechanisms, and prolonged DNA damage can lead to aberrant cellular function and disease progression. Patients with COPD experience persistent airway inflammation and oxidative stress, which can result in the accumulation of DNA damage. Long term DNA damage may disrupt normal cellular function, promoting cellular senescence and apoptosis. Moreover, DNA damage increases the susceptibility to genetic mutations associated with diseases like lung cancer. In individuals with asthma, airway inflammation has the potential to induce DNA damage as well. Inflammatory mediators and reactive oxygen species released by inflammatory cells directly harm DNA while also influencing its repair mechanisms. Repeated instances of DNA damage may impair the regenerative capacity of airway epithelial cells, leading to airway remodeling and decreased pulmonary function.

Early detection, diagnosis, and treatment are the basic principles of disease prevention and treatment. To ensure effective diagnosis and treatment, it is imperative to promptly recognize and assess environmentally driven lung DNA damage. Researchers have utilized various methods of specimen collection to detect and analyze indicators of DNA damage, thereby evaluating the extent of damage based on alterations in these markers to determine treatment efficacy (Table 1).

**TABLE 1:** DNA damage markers from various sources in chronic lung disease.

Disease	Detection indicators/methods	Sample source	References
COPD	Comet experiments	Mice (whole blood)	Hartmann et al (2017)
		Human (peripheral blood)	Da Silva et al (2013)
		Human (peripheral blood lymphocytes)	Dos Santos et al (2022); Ilari et al (2022); Habas et al (2018); Oit-Wiscombe et al (2013)
		Human (peripheral blood monocyte)	Ben Anes et al (2014)
		Human (HASMCs)	Wu et al (2013)
	γH2AX/IF	Guinea pigs' lung tissue	Aoshiba et al (2012)
		Human lung tissue	Aoshiba et al (2012); Pastukh et al (2011)
		Rat lung tissue	Tao et al (2023)
	γH2AX/IF, IHC	Mice lung tissue	Itoh et al (2014); Zhou et al (2015); Karagiannis et al (2012)
	8-OHdG/IF,WB	Human lung tissue	Aoshiba et al (2012); Yang et al (2014)
	8-OHdG/IHC	Mice lung tissue	Araya et al (2019); De et al (2022); Suzuki et al (2017); Liu et al (2018)
		Rat lung tissue	Zhang et al (2018)
	8-OHdG/Elisa	HBE cell	Leclercq et al (2017)
		Human serum	Cao et al (2022); Liu et al (2019)
		Rat serum	Li et al (2017a)
		Guinea pigs plasma	Paul et al (2018)
		Human urine	Igishi et al (2003); Huang et al (2021); Grady et al (2018); Nemoto et al (2014)
		Mice BALF	Ng et al (2014)
		Rat BALF	Li et al (2017b)
		Human EBC	Fireman Klein et al (2019)
	8-OHdG/HPLC-ECD	HASMCs	Wu et al (2013)
Asthma	Comet experiments	Human (peripheral blood leukocyte)	Hasbal et al (2010); Dilek et al (2015); Gaballah et al (2018); Ben Anes et al (2016)
		Human (peripheral blood lymphocytes)	Osman et al (2018)
		HBEpiCs, BEAS-2B, A549	Yuan et al (2020); Zhang et al (2020); Wang et al (2014); Chan et al (2017)
		Mice BALF	Wang et al (2018)
		Human lung tissue	Chan et al (2016)
	γH2AX/IF, WB, IHC	Mice lung tissue	Chan et al (2016); Zhang et al (2020); Wang et al (2018)
		Human lung tissue	Chan et al (2016); Zhang et al (2020)
	γH2AX/WB	Mice bone marrow progenitor cells	Echeverri Tirado et al (2019)
	γH2AX/IF	BEAS-2B	Chan et al (2017)
	8-OHdG/Elisa, IF, IHC	Mice lung tissue	Chan et al (2016); Zhang et al (2020); Chen et al (2021); Zhao et al (2020); Zhu et al (2019); Zhang et al (2019); Rehman et al (2020)
	8-OHdG/Elisa	16HBE	Zhang et al (2020)
		Mice serum	Zhu et al (2019); Rehman et al (2020); Wafriy et al (2023)
		Mice BALF	Chan et al (2016); Zhang et al (2020); Wang et al (2018); Rehman et al (2020); Calciano et al (2018)
		Human urine	Calciano et al (2018); Babadi et al (2022); Kuang et al (2020); He et al (2021)
BPD	Comet experiments	Rat lung tissue	Tong et al (2023); Jin et al (2015)
		Rat AECII	Tong et al (2023); Jin et al (2015)
	γH2AX/IF, WB	Rat lung tissue	Tong et al (2023)
		Rat AECII	Tong et al (2023)
	8-OHdG/Elisa, IHC	Rat lung tissue	Tong et al (2023); Jin et al (2015); Akduman et al (2021); Muramatsu et al (2016); Tayman et al (2021)
	8-OHdG/Elisa	Human serum	Hsiao et al (2017)
		Human urine	Joung et al (2011); Tokuriki et al (2015); Cui and Fu (2022)
		Human TA	Hsiao et al (2017); Hsiao et al (2021)

### 3.1 Lung tissue and cell level

The extent of DNA damage in diseases was assessed based on indices associated with DNA damage in human and animal lung tissues as well as lung cells. Currently, the focus of this research is on employing techniques such as the comet assay, γH2AX detection, and 8-OHdG detection.

#### 3.1.1 Comet experiments

The Comet experiment, also referred to as the single-cell gel electrophoresis experiment, involves subjecting cells to agarose gel cracking, which induces untangling and migration of DNA within the cell in response to an electric field. In case of DNA damage, fragmented DNA segments are eliminated from the nucleus through an electric field, resulting in a comet-like trail formation. By analyzing the shape and length of this comet structure, it becomes feasible to evaluate the extent of cellular DNA damage. Comet experiments are commonly used to study how diseases affect DNA damage. This technique is prized for its speed, simplicity, and sensitivity in quantifying DNA breaks and repairs at the cellular level. Alkaline comet detection identifies both SSBs and DSBs, while the neutral comet detection method exclusively detects DSBs (Yuan et al., 2020). Chan et al. (2016), exposed the human bronchial epithelial cell line BEAS-2B and human alveolar epithelial cell line A549 to HDM and elucidated the direct DNA-damaging effects of HDM on these cells using an alkaline comet assay. Comet assay had been used to reveal DNA damage in human lung tissues and bronchial epithelial cells (HBEpiCs) in models of CS-induced COPD or HDM-triggered asthma (Wu et al., 2013; Zhang et al., 2020; Wang et al., 2014). Multifactorial induction experiments demonstrated an increase in DNA damage in human bronchial epithelial BEAS-2B cells upon exposure to HDM, lipopolysaccharide (LPS), or cockroach allergen extract (CAE). The extent of DNA damage was evaluated by quantifying the proportion of fragmented DNA that migrated at a faster rate than the intact DNA in the Comet assay (Chan et al., 2017). Tong et al. (2023); Jin et al. (2015) employed alkaline comet analysis to assess DNA damage in AECII cells. They observed DNA migration from the nucleus in hyperoxia cells, forming a comet tail, and noted increased nuclear DNA migration within 12 h, with more pronounced differences at 24 and 48 h.

#### 3.1.2 γH2AX

The histone variant γH2AX plays a crucial role in the cellular response to DNA damage, particularly when cells are exposed to ionizing radiation or chemotherapy drugs that induce DNA double-strand breaks. Upon phosphorylation at serine 139, histone H2AX rapidly forms γH2AX, which then accumulates at the site of the break and manifests as distinct focal points representing individual DNA double-strand breaks. After the occurrence of DSBs, H2AX was phosphorylated, forming γH2AX, a reliable indicator of DNA damage. γH2AX also plays a crucial role in signaling pathways involved in DDR and repair. CS, a common cause of chronic lung disease, increased γH2AX expression in the lungs of exposed mice compared to that in normal controls (Zhang et al., 2020; Itoh et al., 2014). The examination of lung tissue samples from COPD individuals revealed the presence of γ-H2AX in cells lining the alveolar walls, a phenomenon that was absent in the control subjects. Moreover, COPD patients who smoke exhibited a significantly higher incidence of γH2AX foci in both type I and II alveolar cells as well as in endothelial cells. Additionally, an increased number of γH2AX foci was found to be associated with processes such as apoptosis, senescence, alterations in pro-inflammatory phenotype, and DNA oxidation (Aoshiba et al., 2012; Pastukh et al., 2011). Lung tissue staining in rats with acute COPD caused by CS exposure and bacterial infection further confirmed that DNA damage triggers COPD (Tao et al., 2023). γH2AX-positive cells were often observed in the lung tissues of CS exposed mice, indicating DNA damage in airway and alveolar epithelial cells linked to COPD development via apoptosis (Zhou et al., 2015).

In a mouse model of naphthalene-induced asthma, the accumulation of γH2AX foci in epithelial cells followed a time-dependent trend. The highest levels were observed at 24 and 48 h, followed by a subsequent decline at 72 h, a phenomenon that is consistent with airway re-epithelization and cellular repair (Karagiannis et al., 2012). Mouse models of OVA or HDM induced asthma were established and lung tissues from human patients with asthma were collected. Immunohistochemistry and protein extraction used to examine the lung tissues of the asthmatic mice. The findings demonstrated a notable increase in both the frequency and protein expression of cells positive for γH2AX in the OVA and HDM groups compared to the control group. The same DNA DSB was evident in the lung tissues of asthma patients, indicating the presence of DNA DSB damage (Chan et al., 2016; Zhang et al., 2020; Wang et al., 2018). Increased expression of γH2AX has also been observed in bone marrow progenitor cells derived from asthmatic mice (Echeverri Tirado et al., 2019). After being exposed to 100 μg of HDM for 6 h, BEAS-2B cells exhibited a noticeable increase in the expression of γH2AX. This suggests that airway cells are directly affected by HDM air allergens, leading to DNA DSBs. Consequently, cell proliferation is hindered, eventually resulting in cell death (Chan et al., 2017).

In the neonatal rat model of hyperoxia-induced BPD, there was a gradual increase observed in the levels of γ-H2AX in both lung tissue and primary AECII from days 3–7 and 14. This increase was significantly greater than that observed in the control group. Similarly, in the AECII cell line, the expression of γ-H2AX in the 24-h BPD model group was higher than that in the control group, and the increase was more significant at 48 h. These findings suggest the progressive accumulation of DSBs in the lungs of BPD rats (Tong et al., 2023).

#### 3.1.3 8-Hydroxy-2′-deoxyguanosine (8-OHdG)

The biomarker 8-OHdG is extensively investigated in the field of oxidative stress, as it primarily arises from the attack of reactive oxygen species (ROS) on guanine bases within DNA. In normal physiological conditions, a minimal amount of 8-OHdG is also produced by the body. However, during oxidative stress, its levels significantly escalate. The quantification of 8-OHdG can serve as an indicator for assessing cellular oxidative stress and is closely associated with the onset and progression of various diseases. Furthermore, as a crucial parameter for evaluating DNA damage caused by environmental pollutants, radiation, drugs, and other factors; 8-OHdG can be employed to determine the extent of such damage inflicted on DNA molecules. Alterations in the level of 8-OHdG may occur during disease progression; thus, rendering it valuable as a surveillance marker. Quantification of 8-OHdG offers a highly sensitive approach for analyzing oxidative DNA damage; thus, 8-OHdG can be used as a promising biomarker for the early detection, evaluation of treatment response, and prognostic assessment of diseases associated with oxidative injury. Hence, 8-OHdG is a clinically significant molecule. Individuals with COPD exhibited significantly higher 8-OHdG levels in the lung tissues and human airway smooth muscle cells (HASMCs) compared to the control, indicating DNA oxidative damage (Wu et al., 2013; Aoshiba et al., 2012). Analysis of lung tissue indicated a 3.1-fold increase in the levels of the 8-OHdG protein among individuals with COPD who smoked compared to non-smoking individuals without any respiratory conditions. This finding suggests the occurrence of oxidative DNA damage, particularly in smoking-associated COPD patients. Additionally, it was observed that smoking-related COPD patients had twice as much 8-OHdG as healthy smokers, highlighting the strong correlation between smoking and elevated levels of 8-OHdG in the lung tissue (Yang et al., 2014). After conducting immunohistochemistry on CS exposed COPD mice, it was observed that the expression of 8-OHdG in the COPD group was significantly elevated compared to that in the control group, which breathed air without any exposure (Araya et al., 2019; De et al., 2022; Suzuki et al., 2017; Liu et al., 2018). Examination of lung tissue samples from PM2.5 exposed COPD rats demonstrated that the expression of 8-OHdG increased with an increase in PM2.5. Additionally, cold stimulation could potentially intensify the impact of PM2.5 on 8-OHdG levels in the lungs of rats with COPD (Zhang et al., 2018). The impact of PM2.5 induced COPD on the expression of 8-OHdG in DHBE cells was found to be more significant than that in NHBE cells (Leclercq et al., 2017).

The levels of 8-OHdG were found to be significantly higher in a HDM and OVA-stimulated mouse asthma model as well as in bronchial epithelial cells (16HBE), than in the control group (Chan et al., 2016; Zhang et al., 2020; Chen et al., 2021; Zhao et al., 2020). Immunohistochemistry revealed a notable increase in the levels of 8-OHdG in the lung airway epithelial cells of asthmatic mice compared with those in their normal counterparts. This observation indicates that the pulmonary epithelium could potentially serve as a focal point for intensified oxidative stress (Zhu et al., 2019; Zhang et al., 2019; Rehman et al., 2020).

8-OHdG levels in newborn rat lung tissue were assessed using an ELISA kit and immunohistochemistry. We observed a significant increase in the 8-OHdG levels in the BPD model group from day 3, with a more pronounced increase on days 7 and 14, indicating early-stage oxidative DNA damage. Furthermore, our results indicate that this oxidative DNA damage progressively accumulates over time (Tong et al., 2023; Akduman et al., 2021; Muramatsu et al., 2016; Tayman et al., 2021). Moreover, 8-OHdG expression in AECII cells significantly increased at all time points (12 h, 24 h, 48 h, and 72 h) following hyperoxia; prolonged hyperoxia led to a gradual increase in the 8-OHdG levels in the lung tissue and cultured AECII cells (Jin et al., 2015).

### 3.2 Blood

After obtaining blood samples from adult, pediatric and animal participants, we performed the comet assay and 8-OHdG detection to assess DNA damage in individuals with a diagnosis of COPD and asthma.

#### 3.2.1 Comet experiments

The alkaline comet assay, performed on whole blood samples from CS-exposed mice, revealed an elevated tail moment, confirming DNA damage (Hartmann et al., 2017). After obtaining a 10 mL peripheral blood from COPD and non-COPD patients, an increase in the damage index of alkaline and neutral comets was observed. Subsequently, when categorizing COPD patients based on their smoking habits, no significant differences were found between individuals with severe smoking-related COPD (i.e., ≥30 cigarettes per day) and those with mild smoking-related COPD (i.e., less than 30 cigarettes per day). However, the DNA damage index was higher in smokers compared to that in non-smokers (Da Silva et al., 2013). The results indicate a strong correlation between COPD severity and DNA damage detected in peripheral lymphocytes (Dos Santos et al., 2022; Ilari et al., 2022; Habas et al., 2018). In another study, a comet assay was used to collect mononuclear cells from peripheral blood, revealing increased DNA damage in individuals with COPD compared to healthy individuals. A significant correlation was also observed between COPD severity and DNA damage (Oit-Wiscombe et al., 2013; Ben Anes et al., 2014).

A study on childhood asthma employed a comet assay to gather venous peripheral blood and separate the white blood cells. This study found increased DNA strand fractures in the mild asthma group compared to those in the moderately persistent asthma group. This observation may be explained by compensatory mechanisms involving enhanced DNA repair and effectively validates the presence of DNA damage in childhood asthma (Hasbal et al., 2010; Dilek et al., 2015). In another study, 60 adult asthma patients were divided into two group, 60 with asthma and 30 healthy age-matched controls. Among the asthma group, 40 had mild-to-moderate asthma, and 20 had severe asthma. Peripheral blood mononuclear cells were collected from all participants for alkaline comet assay analysis. The results revealed significant DNA damage in individuals with asthma compared to controls, with higher levels of damage seen in severe asthma cases (Gaballah et al., 2018). Moreover, a Tunisian study revealed a notable increase in DNA damage among individuals with asthma at different disease stages compared with their unaffected counterparts (Ben Anes et al., 2016). The comet assay revealed that DNA damage was present in lymphocytes from patients with asthma (Osman et al., 2018).

#### 3.2.2 8-OHdG

Several studies have reported a notable increase in serum 8-OHdG levels in models of LPS- and CS-induced COPD in SD rats and guinea pigs (Li et al., 2017a; Paul et al., 2018). In individuals diagnosed with COPD, a negative correlation has been observed between elevated 8-OHdG levels in the bloodstream and lung function. Additionally, a positive association between inflammatory cytokine levels, smoking history, and serum 8-OHdG levels was observed. Logistic regression analysis revealed that increased serum 8-OHdG levels were a risk factor for reduced lung function in patients with COPD. Furthermore, higher serum 8-OHdG concentrations upon admission were associated with longer hospital stays (Yang et al., 2014; Cao et al., 2022). Further analysis showed that 8-OHdG was negatively correlated with FEV1, FEV1%, and FEV1/FVC and positively correlated with C-reactive protein, calcitonin, and neutrophil CD64 levels in the acute exacerbation of COPD (Liu et al., 2019). In a study using pregnant mice with OVA-induced asthma, we observed a significant increase in the expression of 8-OHdG in the OVA-exposed group compared to that in the control group. The expression levels gradually increased with the number of OVA administered. Furthermore, noticeable variations were observed during embryonic development, including changes in body size and reduced growth rate (Wafriy et al., 2023). The levels of 8-OHdG in the serum are significantly elevated in OVA treated asthmatic mice compared to those in normal mice (Zhao et al., 2020; Zhang et al., 2019). Researchers observed a notable increase in the expression of 8-OHdG at birth compared to that in control subjects, demonstrating BPD’s role in lung DNA damage. Subsequent analysis conducted on day 28 postnatally revealed a significant increase in 8-OHdG levels, surpassing those observed in the control group (Hsiao et al., 2017).

### 3.3 Urine

Urine samples from smokers diagnosed with COPD showed increased 8-OHdG levels, which were negatively correlated with FVC, FEV1, and arterial oxygen tension (Igishi et al., 2003). COPD patients spend part of their time at home, and exposure to PM and indoor black carbon (BC) attached to radionuclides may promote the oxidative degradation of DNA in COPD patients, resulting in a higher expression of 8-OHdG than that in the control group (Huang et al., 2021; Grady et al., 2018). However, increased expression of 8-OHdG was detected in the urine of patients with severe COPD following pulmonary rehabilitation. This may be related to the aggravation of DNA damage caused by anaerobic conditions after high-intensity exercise and could serve as a useful indicator for estimating the level of 8-OHdG in the urine of patients with asthma was higher than that in the urine of healthy controls (Nemoto et al., 2014). Additionally, 8-OHdG tended to increase Feno levels and reduce lung function, effectively reflecting the pattern of inflammation involved in oxidative stress (Calciano et al., 2018; Babadi et al., 2022; Kuang et al., 2020; He et al., 2021). Urine samples were collected on days 1, 3, and 7 after the birth of BPD infants. The 8-OHdG level on day 7 reflects the degree of oxidative stress and serves as an independent risk factor for the development of moderate/severe BPD. Furthermore, the level of 8-OHdG on day 3 was significantly correlated with the duration of mechanical ventilation. Evidently, urinary 8-OHdG is an indicator of oxidative damage-associated BPD development and prognosis (Joung et al., 2011). To increase their understanding, the researchers incorporated additional time points and assessed BPD levels in infants by measuring urinary 8-OHdG levels on days 5–8, 12–15, 19–22, and 26–29 postnatally. Infants with moderate BPD exhibited increased urinary 8-OHdG levels compared to those in the no or mild BPD group from days 5–8 postnatally (Tokuriki et al., 2015). In a further study, 8-OHdG levels in infants of the BPD group were significantly elevated on the 7th, 14th, 21st, and 28th days postnatally. Similarly, urine 8-OHdG levels were positively correlated with the duration of mechanical ventilation and oxygen exposure time. BPD development can be predicted as early as the 14th postnatal year (Cui and Fu, 2022).

### 3.4 Bronchoalveolar lavage fluid (BALF)

The BALF method is a relatively mild sampling technique that provides a direct and objective assessment of DNA damage in the lungs.

#### 3.4.1 Comet experiments

After establishing the mouse model of OVA-induced asthma, alkaline comet detection was conducted in BALF to quantify DNA damage. Compared with the control group mice, mice in the OVA-induced asthma group showed elevated DNA damage (Wang et al., 2018).

#### 3.4.2 8-OHdG

**FIGURE 2:**
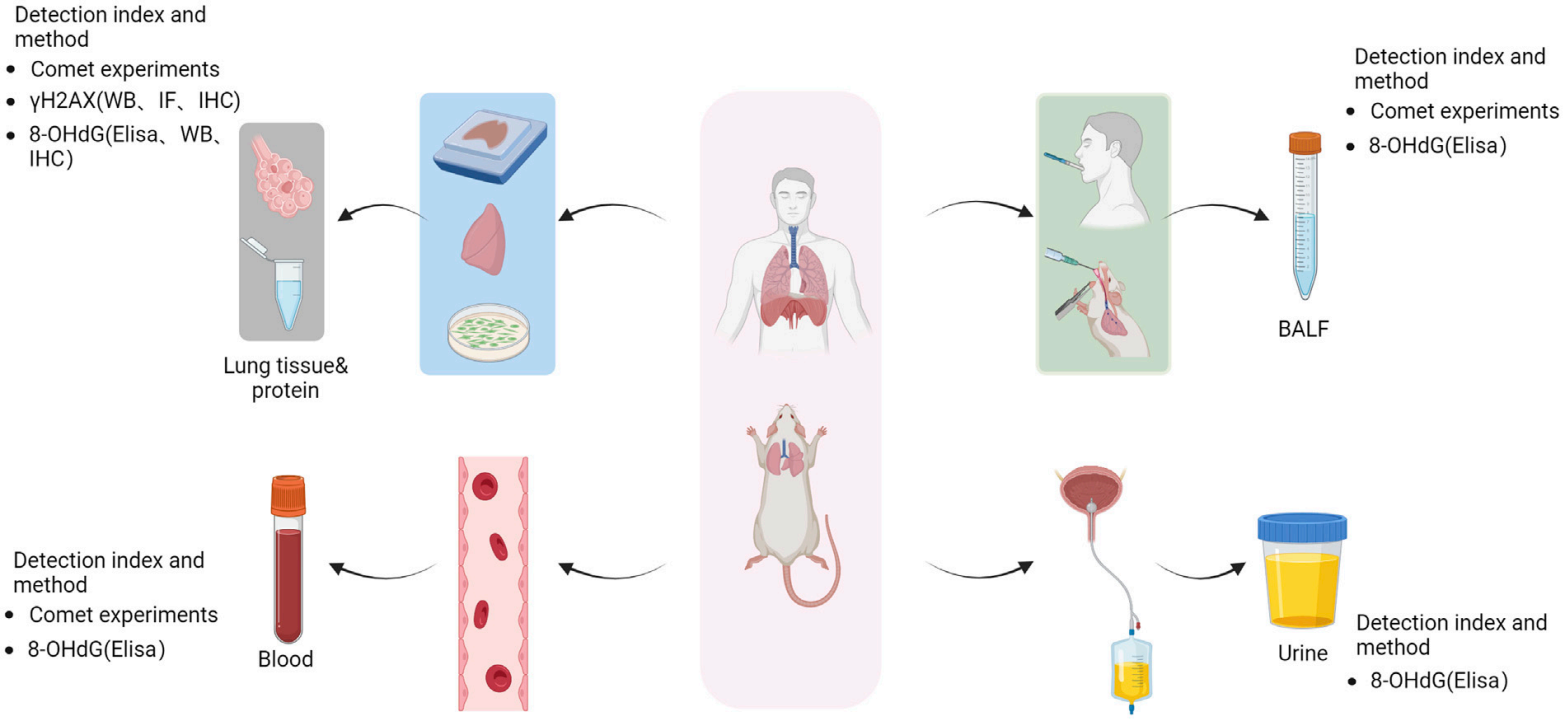
Method for the detection of lung DNA damage. Following lung DNA damage, human and animal lung tissue samples were collected and processed to obtain tissue slices, tissue protein extracts, and cell extracts. The alterations in the levels of γH2AX (WB, IF, IHC) and 8-OHdG (Elisa, WB, IHC) were assessed using comet assays to evaluate the extent of DNA damage. Additionally, blood samples and bronchial lavage fluid were collected for comet tests to assess DNA damage and measure urinary concentrations of 8-OHdG using ELISA. Image generated using Bio-Render.

Expiratory condensation (EBC) is a noninvasive technique that involves collection of exhaled air into a cooling tube. Analysis of EBC samples revealed significantly higher 8-OHdG levels in COPD patients compared to patients in the healthy group (Fireman Klein et al., 2019). In a rat model of LPS-induced COPD and CS, analysis of BALF showed elevated levels of 8-OHdG in the COPD group (Li et al., 2017b). Similarly, in a mouse model of CS-induced COPD, the BALF samples from the COPD group exhibited increased levels of 8-OHdG (Ng et al., 2014). To assess oxidative damage in the airway, 8-OHdG levels were measured in the BALF of a mouse model of OVA-induced asthma, which demonstrated a significant increase in 8-OHdG levels after exposure to OVA. Furthermore, following O_3_ intervention, a significant increase in this index was observed, indicating oxidative DNA damage and exacerbation caused by exposure to O_3_ (Wang et al., 2018; Rehman et al., 2020; Bao et al., 2018). Similarly, in a dust mite-induced mouse asthma model, significantly higher expression levels of 8-OHdG were detected in the BALF than in the control group, ultimately leading to apoptosis (Chan et al., 2016; Zhang et al., 2020). Two cohort studies identified oxidative DNA damage in the lung tissues of children with BPD. Expression of 8-OHdG was assessed through tracheal aspiration (TA) at both 1 day and 28 days postnatally for low-birth-weight infants. Children with BPD exhibited higher levels of 8-OHdG at 1 day post-birth, which increased with age and remained significantly elevated compared to the controls at 28 days post-birth (Hsiao et al., 2017; Hsiao et al., 2021) (Figure 2).

## 4 DNA damage repair and chronic lung diseases

The most perilous form of DNA damage, the primary repair pathways for DNA double-strand breaks encompass non-homologous end-joining and homology-directed repair, which are indispensable for upholding genomic stability. Following a double-strand break (DSB), the non-homologous end joining (NHEJ) pathway promptly recognizes and binds to the damaged ends, initiated by the Ku protein which recruits other proteins to form complexes. Subsequently, a cascade of enzymes facilitates the direct ligation of severed ends without requiring a homologous template throughout the process. This mechanism operates across all cell cycle stages, effectively preventing cellular death or genomic instability-induced mutations by directly rejoining the broken ends. HDR is usually processed by nucleases after DSB to cleave the broken end of DNA, generating a single-stranded break. It then conducts a homologous sequence search to find a sister chromatid or homologous chromosome with a matching end or sequence as a template. This promotes base pairing between the single-stranded DNA and the homologous sequence. Next, the homologous sequence serves as a template for DNA polymerase to synthesize a new strand that fills the gap at the broken end. Finally, ligase connects the newly synthesized DNA strand with the original strand, ensuring accurate repair of DSBs and preventing incorrect connections and mutations from accumulating. This not only maintains genome integrity but also ensures stability.

### 4.1 NHEJ

The NHEJ is the primary mechanism utilized by mammalian cells to repair DSBs. NHEJ is active throughout all phases of the cell cycle and represents a faster and more efficient alternative to homologous recombination (HR) deficiency in this pathway can result in the development of many human diseases. The key intermediaries of NHEJ repair pathways are Ku70, Ku86, DNA ligase IV, XRCC4 (X-ray repair cross-supplement protein 4), Artemis, XLF (XRCC4-like factor), DNA-dependent protein kinase, and the catalytic subunit (DNA-PK).

#### 4.1.1 NHEJ and COPD

Mice lacking Ku86 exhibited increased early aging and mortality as well as selective reductions in the expression of the DNA repair protein Ku86 in the bronchial epithelium of COPD patients and CS-exposed mouse strains. Additionally, XRCC4 inactivation is associated with programmed cell death or apoptosis, and its expression was elevated in COPD patients. Importantly, these cells undergo reparative processes after injury (Da Silva et al., 2013). Increased 53BP1 expression within the NHEJ repair pathway was observed in ATII cells from COPD patients and mice with CS-induced COPD. Another study has reported high levels of DSBs in ATII cells obtained from COPD patients. Conversely, proteins involved in DNA damage repair (such as 53BP1, DNA ligase IV, XRCC4, XLF, and γH2AX) demonstrated reduced expression; however, there were no differences in RAD51 (HR pathway intermediary) when compared to the control group. These findings suggest that the HR pathway is ineffective in DNA damage repair (Messier et al., 2013; Kosmider et al., 2019). The variations observed in related proteins within the NHEJ pathway across different experiments may be attributed to distinct cellular states at various disease time points, indicating different primary modes of repair; further research is necessary.

#### 4.1.2 NHEJ and asthma

The key DNA repair protein, Ku70, plays multiple roles, including those in VDJ recombination, telomere maintenance, and regulation of cell homeostasis. Studies have revealed a reduction in the expression of Ku70 in the airway epithelia of asthmatic mice. Overexpression of Ku70 has been shown to alleviate airway hyperresponsiveness, inflammation, and epithelial fibrosis while reducing TGF-β levels. However, it did not affect IL-13 levels or goblet cell metaplasia (Rehman et al., 2021). DNA-PKCs are essential for lymphocyte maturation during mammalian development as they aid DNA repair; their functions are well-studied in mature lymphocytes. For instance, Mirsha et al. found HDM-induced DNA-PKC activation in a mouse asthma model, which is crucial for Th2-mediated inflammation (Mishra et al., 2015). Furthermore, in murine models of OVA- and HDM-induced asthma, DNA-PKCs play a crucial role in regulating the differentiation of CD4^+^T cells into Th1 and Th2 subsets by modulating the expression of the transcription factors Gata3 and T-bet. Additionally, DNA-PKC gene hybridization revealed that constructing heterozygous genes reduced eosinophilia, mucus hypersecretion, Th2 cytokine production, OVA-specific IgEs, and airway hyperresponsiveness in asthmatic mice (Ghonim et al., 2015). Both studies demonstrated that treatment with DNA-PKC inhibitors alleviated the severity of asthma-associated symptoms, possibly because of their ability to reduce NHEJ repair activity, which exerts an immunosuppressive effect, leading to decreased sensitization.

#### 4.1.3 NHEJ and lung development

Previous studies indicate that KU70-deficient mice show premature aging features likely due to increased DNA damage because of impaired DNA repair. Additionally, KU70-knockout mice display lung development issues, increased apoptosis, and pulmonary diseases like emphysema and pulmonary artery occlusion by 3 months of age. These lung problems may lead to secondary health issues, including heart failure, contributing to reduced survival in KU70-knockout mice (Ngo et al., 2015). While some existing studies have indicated the potential implications of NHEJ impairment in mammalian nervous system development, further investigation is warranted to elucidate its impact on lung development and NHEJ.

### 4.2 HDR

While HDR is known for its precision, it has limited efficiency. HDR utilizes homology templates for error-free DNA repair. Key repair proteins, including BRCA1/2, BLM, DNA2, EXO1, MRN (MRE11, RAD50, NBS1), RPA, and RAD51/52, are involved in this process. BLM and DNA2 work in concert to excise DNA in an ATP-dependent manner. RPA unwinds the BLM-bound DNA and enforces 5′→3′ excision polarity for DNA2. MRN recruits BLM for action, and EXO1 participates in excision when stimulated by BLM, MRN, and RPA. BLM enhances EXO1 affinity for termini, while MRN recruits and enhances its function. The termination of human DNA repair remains under investigation by researchers.

#### 4.2.1 HDR and COPD

BRCA1 is a major protein involved in the HDR pathway, which repairs damaged DNA. In CS-exposed mouse COPD models, the levels of the apoptotic proteins BIM and HDR protein BRCA1 were increased, and the expression of BIM and BRCA1 was inversely proportional to that of miR-24-3p (Nouws et al., 2021). Contrary to previous findings suggesting the ineffectiveness of the HDR pathway in repairing DNA damage in COPD cells owing to the lack of changes in RAD51 expression, it becomes evident that this conclusion is inappropriate. The elevated levels of BRCA1 and apoptosis observed in COPD may be attributed to the impaired efficiency of the HDR pathway and an inadequate DDR response. Currently, there are limited studies investigating the relationship between the HDR pathway and COPD; further exploration is required to elucidate the specific mechanism.

#### 4.2.2 HDR and asthma

RAD50 is constitutively expressed in various tissues and actively participates in the repair of double-stranded DNA breaks. Chen et al. reported an association between RAD50 polymorphisms and asthma development in a Chinese population (Chen et al., 2015). However, subsequent studies have demonstrated that variants rs2244012 and rs6871536 within RAD50 were not significantly associated with childhood asthma in Northeast China (Li et al., 2016). These findings emphasize the necessity for a more comprehensive selection of gene variants to assess the broader risk of childhood asthma, enabling the identification of key genetic markers that can accurately predict individual susceptibility to asthma in children and facilitating further exploration of the underlying mechanisms.

#### 4.2.3 HDR and lung development

**FIGURE 3:**
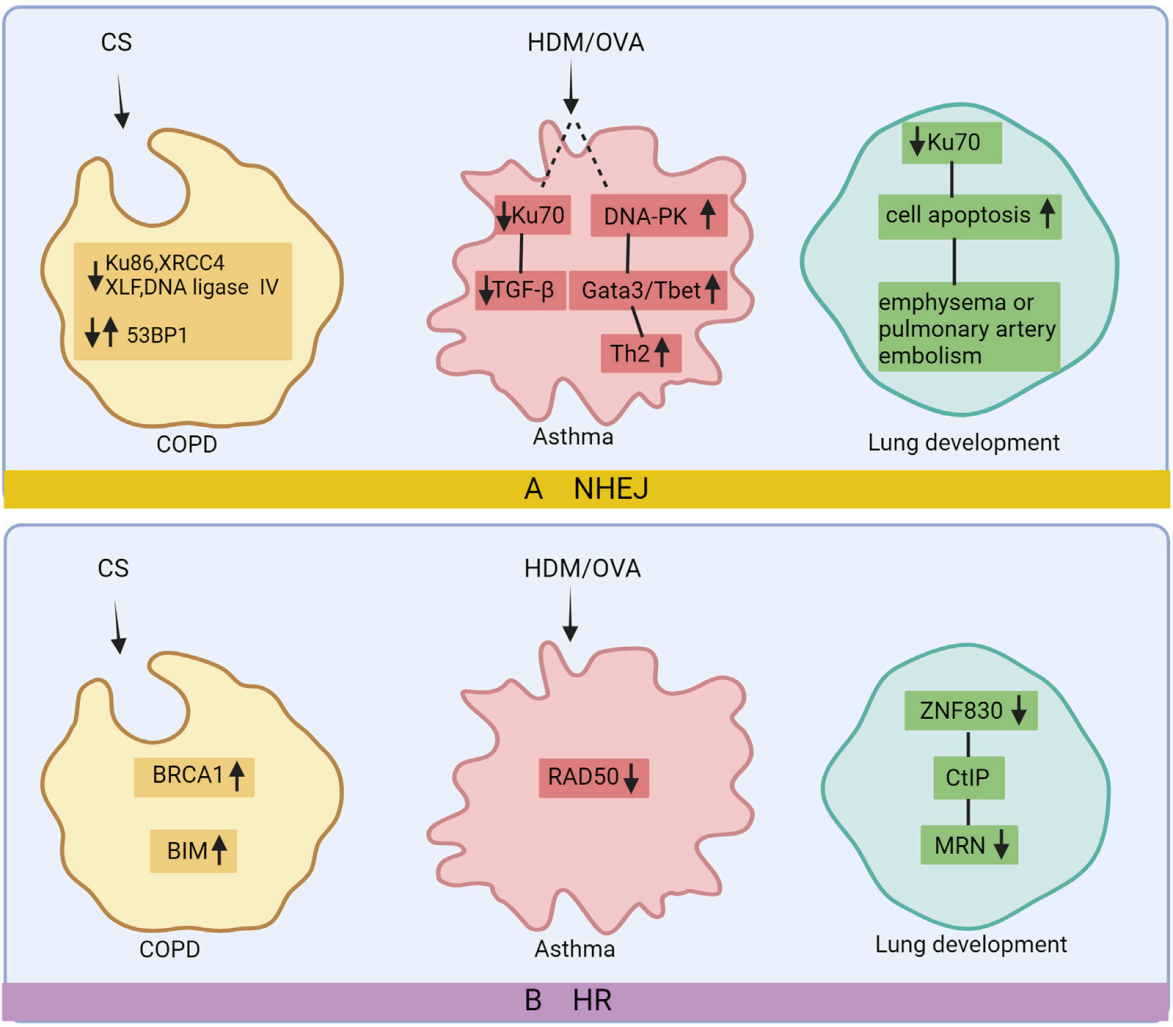
DNA repair after damage in chronic lung disease. In COPD’s non-homologous end joining (NHEJ) pathway, there is a reduction in Ku86, XRCC4, DNA ligase IV, and XLF levels, while 53BP1 expression varies. In the homologous recombination (HR) pathway, BRCA1 and BIM expression increases. Asthma’s NHEJ pathway exhibits downregulated Ku70 and upregulated DNA-PK, leading to elevated Gata3/T-bet and Th2 levels. RAD50 expression decreases in asthma’s HR pathway. Reduced Ku70 contributes to increased apoptosis, emphysema, and PA occlusion in lung development via NHEJ, while decreased ZNF830 in the HR pathway results in lower CtIP-MRN levels and lung dysplasia. Image created with Bio-Render.

Previous studies elucidated the involvement of ZNF830 in HDR-mediated DNA repair and its direct interaction with CtIP, a key player in promoting MRN-mediated DNA end resection. In addition, ZNF830 deficiency results in developmental defects (Chen et al., 2018). Currently, there is limited research on the role of HDR in lung development and BPD pathogenesis, which are relatively complex phenomenon. Further investigation and confirmation are required to explore whether HDR contributes to lung development or BPD and to unravel its underlying mechanism (Figure 3).

## 5 Potential treatments for relieving chronic lung disease by intervening in DNA damage

Treatments for chronic lung disease associated with DNA damage involve mitigating pulmonary DNA damage and enhancing the DNA repair capacity in the lungs. Nonsteroidal anti-inflammatory drugs (NSAIDs) are widely used to suppress inflammation. Aspirin, a non-steroidal anti-inflammatory drug, widely used for its anti-inflammatory properties is associated with several systemic side effects including gastro-intestinal discomfort. Inflammation can be mediated by pro-inflammatory cytokines and, along with various other host factors eventually give rise to edema at the inflamed site. The results showed that particle size (90 nm) populations of nano-emulsion preparations of aspirin compared to an aspirin suspension (363 nm), the aspirin nano-emulsion further reduced the expression of inflammatory factors and edema at the site of inflammation (Subramanian et al., 2008). In the past decade, significant advancements have been made in the field of research and development of nano therapy. When aspirin nanosuspension (ASP N) was administered to patients with COPD and asthma, researchers observed a notable reduction in DNA damage within their lymphocytes, indicating that nano therapy effectively enhanced the drug’s efficacy (Najafzadeh et al., 2016). The inflammatory and oxidative responses in COPD rats exposed to PM2.5 and cold stimulation were enhanced, while the expression of 8-OHdG was significantly decreased in the group treated with valsartan under conditions of cold stress. Valsartan may partially alleviate DNA damage caused by cold stress and exposure to PM2.5 in COPD rats (Liu et al., 2018). Treatment with bulk quercetin and nano quercetin resulted in a decrease in lymphocytes among healthy individuals and those diagnosed with COPD. This was accompanied by a significant reduction in DNA damage, as demonstrated by comet measurements (Habas et al., 2018). The protective effects of ALDH3A1, a member of the ALDH superfamily, have been observed in bronchial epithelial cells affected by COPD. It regulates the expression of FANCD2, thereby protecting against DNA damage and cytotoxicity (Jang et al., 2014). The scientists have made a significant discovery: the absence of CXCR2 in mice offers protection against COPD caused by CS. This protective effect is achieved by shielding lung cells from DNA damage and aging. Targeting the CXCR2 receptor, which is primarily located on neutrophils and essential for their immune-modulating activity through receptor antagonism, presents a pharmacological approach to mitigate the potentially detrimental effects of excessive neutrophil infiltration into the lungs.

Previous studies have demonstrated that mice lacking CXCR2 are resistant to lung injury, highlighting its potential as a therapeutic target for preventing DNA damage during development (Lerner et al., 2016). Molecular hydrogen (H_2_) has been identified as a targeted preventive and therapeutic antioxidant that effectively interacts with hydroxyl radicals. After the introduction of H_2_, the COPD group induced by CS exhibited a significantly lower number of 8-OHDG-positive cells than the CS and COPD group, suggesting that molecular H_2_ holds promise as an innovative therapeutic approach for various ailments, including COPD. The supply of molecular H_2_ to drinking water is much more convenient and safer than other routes of administration, and molecular H_2_ may be a novel and promising therapeutic approach (Suzuki et al., 2017). In addition, ursolic acid, which is naturally present in various plants, such as apple peel and herbs, has shown promise in mitigating DNA damage in lung tissue affected by COPD. This is achieved through the regulation of the PERK and Nrf2 pathways, leading to a decrease in the levels of 8-OHdG compared to those in the group with COPD (Lin et al., 2017).

Chinese medicine research has suggested that Tong Sai granule can improve lung function by regulating the MAPK-SIRT1-NF-κB pathway, alleviating inflammation caused by CSE/LPS in COPD lung tissue and mitigating cellular senescence in rats (Tao et al., 2023). The formulation of recuperating lung decoction (RLD) is based on the principles of classical Chinese medicine. In an experimental rat model with LPS and CS induced COPD, RLD showed effectiveness in reducing airway inflammation, indicating its potential as an agent with anti-inflammatory properties. Furthermore, RLD significantly reduced emphysema and inflammation caused by LPS and CS exposure. These beneficial effects could be attributed to the inhibition of the ERK/Nrf2 and MAPK/AP-1 signaling pathways while modulating gamma-glutamyl cysteine synthetase activity to alleviate lung injury induced by oxidative stress (Li et al., 2017a; Li et al., 2017b).

To test the effect of antioxidant efficacy at treating asthma, BEAS-2B cells were exposed to exogenous glutathione or catalase during HDM exposure. These antioxidants eliminated ROS and reduced DNA damage. Catalase lowered the ROS levels and reduced HDM-induced DNA damage in BEAS-2B cells. Apocynin also reduced HDM-induced DSBs in these cells, as observed by decreased γH2AX expression. Antioxidants have potential for mitigating DNA damage in HDM-induced asthma (Chan et al., 2017). Researchers have emphasized the significance and effectiveness of exogenous antioxidants in protecting the lung cells from the genotoxic effects of airborne allergens. This discovery revealed new possibilities for asthma treatment using antioxidants. Resveratrol (RES), an antioxidant found in red grape skin, red wine, and peanuts, possesses anti-apoptotic properties, and influences various biological processes. Evaluation of DNA damage using comet assay confirmed RES’s ability to alleviate HDM-induced DNA damage in bronchial epithelial cells affected by asthma. Additionally, experimental evidence suggests that RES provides protection against HDM-induced apoptosis (Zhang et al., 2020).

**FIGURE 4:**
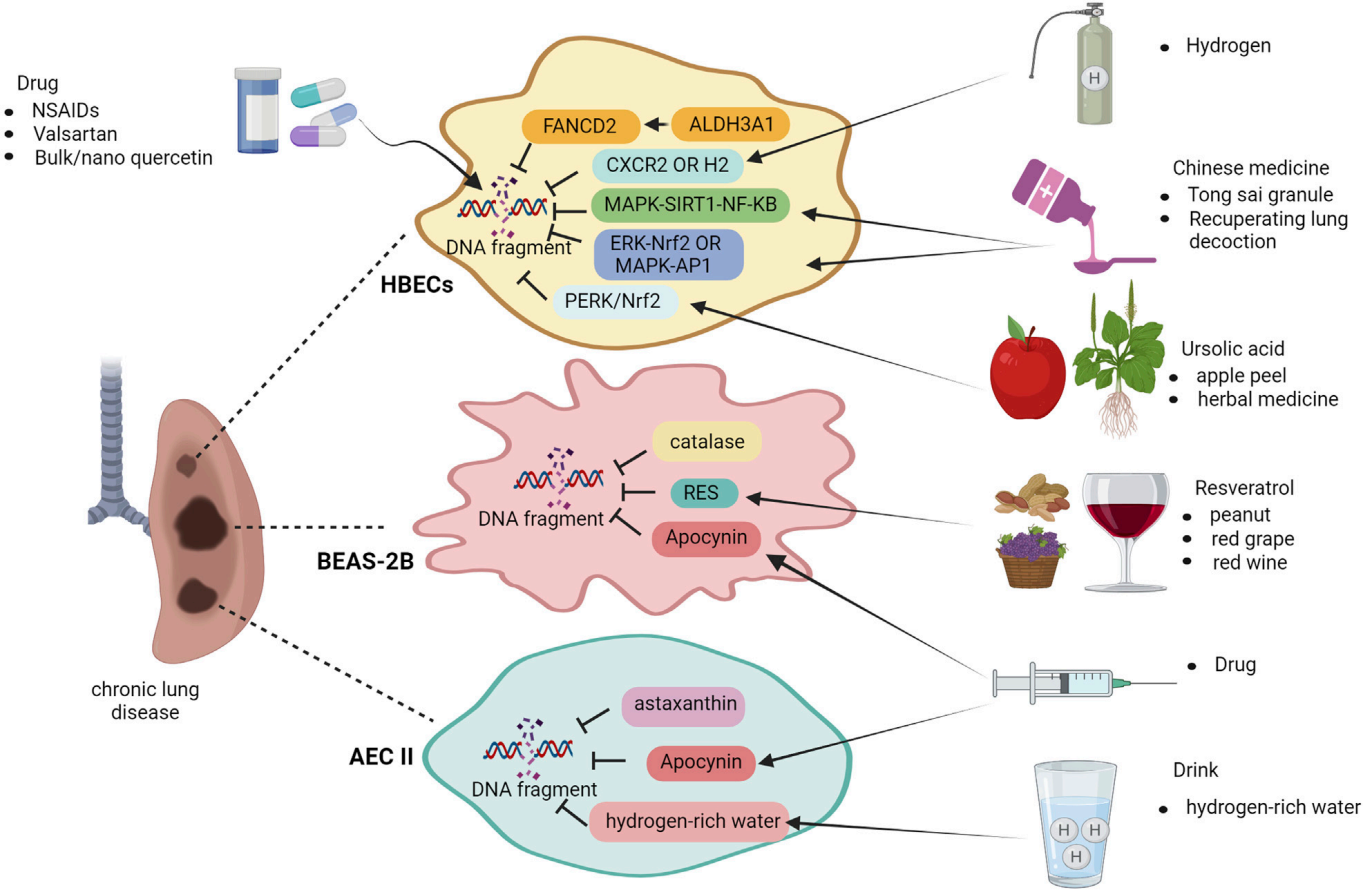
Treatment of chronic lung disease after DNA damage. Non-Steroidal Anti-Inflammatory Drugs (NSAIDs), valsartan, bulk and nano quercetin, H_2_ molecules, traditional Chinese medicine Tong sai granule, and RLD offer therapeutic relief in case of COPD-induced lung DNA damage. Ursolic acid activates the ERK/Nrf2 pathway. In asthma, apocynin, resveratrol, and H_2_O_2_ reduce ROS levels and apoptosis. Apocynin, astaxanthin, and H_2_-rich water mitigate apoptosis and DNA damage in BPD lungs for positive therapeutic outcomes. Image created with Bio-Render.

The effects of astaxanthin, a compound with anti-inflammatory, antioxidant, and antiapoptotic properties, were examined in a rat model of bronchopulmonary dysplasia (BPD) induced by hyperoxia and LPS. Administration of astaxanthin resulted in decreased expression of 8-OHdG in the lung tissues of BPD rats compared to that in the BPD group. This reduction can be attributed to the enhanced antioxidant activity of astaxanthin, which effectively counteracts oxidative stress and protects DNA and proteins from damage. Additionally, its antioxidant properties contribute to the inhibition of apoptosis and protection against lung injury (Akduman et al., 2021). The expression of NOX4/DUOX1, which is responsible for promoting ROS production, is effectively inhibited by astaxanthin (AST) in cigarette smoke extract (CSE)-induced COPD, leading to a reduction in DNA damage and apoptosis (Tang et al., 2023). AST may be a potential protective agent against CSE-associated lung disease and deserves further study. Apocynin, also known as 4-hydroxy-3-methoxyphenylacetone or acetyl amphetamine, occurs naturally in the body and effectively reduces the production of superoxide anions (O^2−^) by activated neutrophils and macrophages. This inhibition leads to a decrease in ROS generation. In a neonatal rat model of postnatal hyperoxia- and LPS-induced BPD, treatment with apocynin significantly decreased the expression of 8-OHdG in the lung tissue compared to that in the BPD group on day 14. These findings suggest that apocynin exerts potential therapeutic effects by reducing cellular apoptosis and damage (Tayman et al., 2021). Researchers are currently examining the potential benefits of administering hydrogen-rich water to pregnant rats in BPD modeling experiments to investigate whether prenatal maternal intervention can be a preventive measure for BPD in infants. This is because hydrogen-rich water has been reported to have minimal side effects. The findings of this study revealed that newborn rats with maternal consumption of hydrogen-rich water had significantly lower levels of 8-OHdG in their lung tissues than those in the BPD group (Muramatsu et al., 2016). These results suggest that the incorporation of hydrogen-rich water into the diet of high-risk infants may be an effective strategy for preventing BPD (Figure 4).

## 6 Conclusion

The present study delves into the intricate association between DNA damage and chronic lung disease. By summarizing the research findings, we have unearthed compelling evidence of DNA damage in individuals afflicted with chronic lung disease, which exhibits a close correlation with disease severity, progression, and other contributing factors. These significant discoveries offer valuable insights for further comprehending the pathogenesis of chronic lung disease. These instances of DNA damage can be attributed to prolonged exposure to detrimental environmental factors such as air pollution and smoking, resulting in heightened oxidative stress that subsequently triggers DNA damage. Moreover, chronic inflammatory response may also exert a substantial influence on the occurrence and development of DNA damage. The accumulation of DNA damage can impact the normal functioning of lung cells, including cell proliferation, differentiation, and apoptosis, thereby facilitating the progression of chronic lung diseases. These findings imply that DNA damage may serve as a pivotal factor in the development of chronic lung disease.

By monitoring and repairing DNA damage, novel strategies for treating chronic lung disease are anticipated to be provided. It can also be speculated that by intervening in the repair process of DNA damage, it might be possible to decelerate the progression of chronic lung disease. For instance, developing novel types of antioxidants or anti-inflammatory drugs to mitigate the occurrence of DNA damage; or utilizing gene therapy technology to enhance the DNA repair capacity of lung cells. Future research should aim to develop long-term follow-up studies to observe the dynamic relationship between DNA damage and the progression of chronic lung disease, as well as investigate the impact of intervening in DNA damage on disease prognosis. Additionally, interdisciplinary research can be strengthened by integrating knowledge and technology from various fields such as biology, medicine, physics, etc., to collectively explore the intricate association between DNA damage and chronic lung disease. For instance, advanced imaging technology can be utilized for real-time monitoring of DNA damage within lung cells. This paper provides a summary of elevated levels of DNA damage biomarkers detected in diverse samples obtained from patients with COPD, asthma, and BPD. In the realm of DNA damage and chronic lung disease investigation, continuous innovation is required regarding research methods and techniques to enhance efficiency and accuracy. It is worth exploring the development of a novel DNA damage detection kit that enhances sensitivity and specificity or leveraging artificial intelligence technology for analyzing extensive clinical data to identify potential disease markers.

The expectation is that in the future, cooperation and exchanges between disciplines can be strengthened, and even more international collaboration and exchanges can be shared to mutually exchange research results and experiences, thereby jointly promoting the development of this field. Currently, there are numerous unresolved issues within this domain that necessitate further exploration. It is hoped that this study will inspire increased attention from researchers towards this area so they may collaborate in providing more effective strategies for prevention and treatment of chronic lung diseases.
